# A Landscape View of Agricultural Insecticide Use across the Conterminous US from 1997 through 2012

**DOI:** 10.1371/journal.pone.0166724

**Published:** 2016-11-30

**Authors:** Timothy D. Meehan, Claudio Gratton

**Affiliations:** Department of Entomology and Great Lakes Bioenergy Research Center, University of Wisconsin-Madison, Madison, Wisconsin, United States of America; Chinese Academy of Agricultural Sciences, CHINA

## Abstract

Simplification of agricultural landscapes is expected to have positive effects on many crop pests and negative effects on their natural enemies, potentially leading to increased pest pressure, decreased crop yield, and increased insecticide use. While many intermediate links in this causal chain have empirical support, there is mixed evidence for ultimate relationships between landscape simplification, crop yield, and insecticide use, especially at large spatial and temporal scales. We explored relationships between landscape simplification (proportion of a county in harvested cropland) and insecticide use (proportion of harvested cropland treated with insecticides), using county-level data from the US Census of Agriculture and a variety of standard and spatiotemporal regression techniques. The best model indicated that insecticide use across the US has increased between 1997 and 2012, was strongly dependent on the crops grown in a county, increased with average farm income and size, and increased with annual growing degree days. After accounting for those variables, and other unidentified spatial and temporal structure in the data, there remained a statistically significant, moderate, positive relationship between insecticide use and landscape simplification. These results lend general support to the causal chain outlined above, and to the notion that a landscape perspective is useful for managing ecosystem services that are provided by mobile organisms and valuable to agriculture.

## Introduction

The widespread replacement of speciose, perennial, seminatural habitats with annual monocultures (hereafter, ‘landscape simplification’) is expected to increase the connectedness of agricultural landscapes for many crop pests and decrease the amount of habitat for the natural enemies of crop pests. These changes are expected to increase colonization rates and decrease predation rates for crop pests, leading to higher pest pressure on crops. If this chain of logic holds, then increases in pest pressure are expected to either decrease crop yield or, perhaps more likely, increase the use of insecticides to prevent yield reductions [1–5].

Empirical support for this logical progression has been mixed. The supposition that landscape simplification increases colonization by crop pests is grounded in ecological theory [6], though the idea has not been thoroughly explored at the landscape scale outside of a modeling context [7,8]. In contrast, there are many examples of pests that rely on host plants in seminatural habitat [9,10], so landscape simplification could actually have negative consequences for these pests [11]. The general assumption that landscape simplification reduces the diversity and abundance of natural enemies is well supported [3,12,13]. However, it is not unusual for highly mobile natural enemies to be seasonably abundant where there is abundant prey, in areas of dense crop production [14]. The idea that landscape simplification reduces the parasitism or removal rates of crop pests has been supported by several sentinel prey studies [15–18]. Unfortunately, pest removal metrics from these studies often take the form of uncalibrated indices, so it is not always clear if these removal rates have any relationship with actual pest damage to crops. The abundance of crop pests, a net result of pest colonization and natural enemy removal rates, and a correlate of pest pressure, is often, but not consistently, negatively related to landscape simplification [3,5,19]. Finally, while a major goal of integrated pest management programs is to base insecticide application on observed pest pressure [20], these programs are not universally adopted, and it is not clear how tightly these two variables are connected. Given all these contingencies, it is not clear if the logical links between landscape characteristics and agronomic outcomes, such as crop yield and insecticide use, describe a hypothetical exception or a general rule.

Recently, Meehan et al. [5] showed that landscape simplification was positively related to crop pest abundance, and positively associated with insecticide use, across the Midwestern US during 2007. In that study, landscape simplification was represented by the proportion of a county in harvested cropland. Conversely, the proportion of a county in seminatural habitat had a negative relationship with insecticide use. Crop pest abundance was represented by the abundance of several aphid species tracked by a regional pest-monitoring network [21]. Insecticide use was represented by the proportion of harvested cropland in a county treated with insecticide, from the US Census of Agriculture (COA) [22]. Several subsequent studies passed over intermediate relationships, focusing solely on the ultimate relationship between landscape simplification and insecticide use. This is partly because data on pest abundance is relatively difficult to attain for large spatial extents, and because indices of both landscape simplification and insecticide use can be derived from the COA, which has been conducted across the US for several decades.

Results from subsequent studies have varied. Larsen [23] found that the relationship between landscape simplification and insecticide use varied in both strength and direction in the Midwestern US between 1987 and 2007. Meehan and Gratton [24] showed that much of the apparent variation could be attributed to spatial structure in insecticide use data. When using spatial econometric and geostatistical modeling techniques, the relationship between landscape simplification and insecticide use was consistently positive in the Midwest from 1997 through 2007 [24]. Yang et al. [25] considered different metrics of landscape simplification, and their association with insecticide use, using spatial econometric methods. They found that decreased crop diversity and increased corn production was associated with increased insecticide use in the Midwestern US from 1997 through 2012. However, in other parts of the US outside the Midwest, the association between crop diversity and insecticide use was positive, while that between corn acreage and insecticide use remained positive. It is possible that a positive association between crop diversity and insecticide use outside the Midwest reflected high insecticide application rates in areas of diverse fruit and vegetable production [5,23,24,26]. This potential source of confounding was not evaluated. Most recently, Larsen et al. [27] suggested that the relationship between landscape simplification and insecticide use varied across years and agricultural regions of the US. While mostly focused on spatial and temporal heterogeneity, Larsen et al. [27] also evaluated a general model that considered the overall association between landscape simplification and insecticide use across all years and regions. They reported no relationship between these two variables, but noted that conclusions from their study could have been influenced by spatial structure in the data not accounted for by their statistical methods.

In summary, the current literature suggests that, notwithstanding some spatial and temporal variation, there generally has been a positive association between different metrics of landscape simplification and insecticide use in the Midwestern US between 1997 and 2012 [24,25]. However, the current literature also suggests that this positive relationship cannot be generalized from the Midwest region to the conterminous US [25,27]. For reasons described above, it is not clear if the lack of generality at the national scale is due to analytical decisions related to omission of important crop composition variables or treatment of spatial and temporal structure in data. In the present study, we assessed a general relationship between landscape simplification and insecticide use across the conterminous US between 1997 and 2012. The goal of this study was to assess support for an overall relationship between landscape simplification and insecticide use at a national scale (1) after accounting for several important variables related to crop composition, farm economics, and climate and (2) using different analytical methods that complement those of recent studies.

## Materials and Methods

### Data collection and preprocessing

The objective of this analysis was to determine if, after accounting for spatial variation in crop composition, farm economics, climate, and other unidentified spatial and temporal effects, there was evidence for a general positive relationship between landscape simplification and insecticide use between 1997 and 2012 across agricultural landscapes of the conterminous US. Spatial data on insecticide application, crop composition, farmer income, farm size, and landscape simplification were acquired from the COA through the National Agricultural Statistical Service (NASS) of the US Department of Agriculture (USDA) [22].

As in other recent studies, insecticide application was represented by relative insecticide use, a county-scaled index calculated as the total number of hectares treated with insecticide divided by the total number of harvested hectares per county [5,23–25,27]. To be considered treated, a parcel of land must have had insecticide applied at least once during the growing season. Previous studies have shown that, although it is an index, relative insecticide use is proportional to the mass of active ingredient applied to farmland [5,23].

Different crop types regularly receive different amounts of insecticide. This is because pests impact the yields, aesthetic characteristics, and monetary value of crops differently [28]. Thus spatial variation in insecticide use can potentially arise from spatial variation in crop composition. To account for this possibility, crop composition variables were included in this analysis, including the proportions of harvested cropland in a county planted in (1) corn for grain and silage, (2) cotton, orchards, and vegetables, and (3) soybean and wheat [5,23,24,27].

The decision to apply insecticide may depend on additional economic factors related to risk tolerance or the capacity of farmers to afford and apply insecticides [28]. Previous analyses have demonstrated that spatial variation in insecticide use is positively related to farmer net income [5,23,24,27]. For this analysis, farmer net income was calculated as the total net income from farming activities (actual income, not adjusted for inflation) divided by the number of hectares of harvested cropland per county [5,24]. Larsen et al. [27] recently showed that farm size was also an important predictor of insecticide use. Suggested mechanisms behind this association included economic mechanisms, such as reduced relative costs of insecticide purchase or application related to economies of scale, and ecological mechanisms related to field size and farm-scale habitat heterogeneity [27]. For this analysis, average farm size was calculated as the number of hectares of harvested cropland divided by the number of farm operators in a county.

Landscape simplification was represented by the proportion of all land in a county that was in harvested cropland [5,23,24,27]. Previous studies have shown that this proportion is closely related to other metrics of agricultural landscape simplification. For example, it is strongly negatively correlated with the proportion of seminatural habitat (unharvested grasslands, woodlands, and wetlands), and strongly positively related to both the average size and the connectivity of cropland patches in the Midwestern US [5].

All of the COA data described above was acquired from the USDA NASS Quick Stats Database, which provides COA data from 1997 through 2012, using an application programming interface (API, described at http://quickstats.nass.usda.gov/api). After data were downloaded, several data cleaning steps were necessary. Given the focus on the conterminous US, the first step in data cleaning was to remove data associated with Alaska and Hawaii. The second step involved dealing with censored data. Preservation of participant anonymity is an important consideration when reporting results from the COA. Thus, county metrics such as income and insecticide use are not reported when derived from a single farm. In this case it is necessary to (1) drop the county from the analysis, (2) impute a likely value, or (3) enter a zero, with the justification that the value for the metric would be relatively low given it was derived from a single farm. For this analysis, when a value for a metric was not reported, an average value for a county over the four different censuses was imputed [24]. In the event where a metric was not reported for any of the four possible years, a value of zero was entered [5,23,27]. The third data processing step involved removing counties from the analysis with less than 3% of the total county land in harvested crops. This step reflects the main objective of this study, to evaluate the consequences of simplification in agricultural landscapes. Similar studies have shown that general conclusions are not affected by these types of data processing steps [23,24].

Recent work suggests that spatial variation in insecticide use also may be related to climatological factors, such as temperature, which could influence the biology of pests, their hosts, and their natural enemies [25,27]. Thus this study included cumulative annual growing degree days (GDD) per county as an agriculturally relevant measure of temperature. There are many ways to calculate GDD, all of which yield inexact indices that approximate the effects of temperature on growth [29]. Here, cumulative annual GDD was the sum of monthly GDD over 12 months. GDD for each month was the product of average daily GDD multiplied by 30.4 days. Average daily GDD was calculated using the average daily minimum (T_min_) and maximum (T_max_) temperatures for a month, a lower base temperature of 8°C (T_lower_), an upper threshold temperature of 32°C (T_upper_), and the equation [(T_max_ + T_min_) / 2] − T_lower_, where T_min_ was set to T_lower_ when lower than T_lower_, and T_max_ was set to T_upper_ when higher than T_upper_. Average daily GDD values less than zero were set to zero during summation. Monthly temperature data for the conterminous US from 1997 through 2012 came from the PRISM Climate Group [30].

### Statistical analysis

The main objective of the statistical analysis was to evaluate associations between insecticide use, landscape simplification, and other covariates using a diverse set of approaches to deal with possible spatial and temporal autocorrelation in model residuals. This residual autocorrelation, noted in similar studies [5,24,25], could arise for two distinctly different reasons. First, residual autocorrelation could indicate that an important spatially- or temporally-structured pattern or process is not captured by the variables in a model [31–33]. In this case, one common strategy is to add spatial or temporal fixed effects that allow model intercepts to vary according to discrete spatial or temporal units [34]. Another common strategy is to model the unexplained structure in the residuals with spatially or temporally structured random intercepts [32,35]. In contrast, residual autocorrelation could indicate that the effect of at least one independent variable is not stationary, where the strength or direction of the effect varies considerably over space or time [36,37]. In this case, one common strategy is to add interactions between spatial or temporal fixed effects and the putative nonstationary variable [27]. Another common strategy is to include spatially- or temporally-structured random slopes for nonstationary variables [38,39]. A final option is to simply accept spatially or temporally autocorrelated model residuals. However, residual autocorrelation indicates lack of independence in statistical replicates, and can be accompanied by biased parameter estimates and unreliable hypothesis tests [40–45]. The general approach in this analysis was to compare the performance of six models that varied in how they did, or did not, deal with spatially and temporally structured residuals, and then to draw inferences based on a best model or set of best models.

We modeled the data using 6 Bayesian linear models. All 6 models were specific cases of a more general group of latent Gaussian models [46,47]. The latent Gaussian models in this analysis took the general form:

$$\begin{array}{c} y_{it}=Norm\left(\mu_{it},\;\sigma^{2}\right),\\ \mu_{it}=g\left(\mu_{it}\right)=\eta_{it},\\ \eta_{it}=\beta_{0}+\sum_{m=1}^{M}\beta_{m}x_{mit}+\;\sum_{l=1}^{L}f_{l}\left(z_{lit}\right), \end{array}$$ (1)

where *y*_*it*_, the response at county *i* during year *t*, was normally distributed with mean *μ*_*it*_, which was related to a linear predictor *η*_*it*_ through a link function *g*(*μ*_*it*_). In this study, all six models employed an identity link function and Gaussian error distribution. The linear predictor comprised three general components. The first component was a scalar, representing the overall model intercept *β*_0_. Within the second component, *β* was a set of *M* coefficients that quantified the influence of *M* fixed effects *x*. Together, components one and two can be considered a generalized linear model (GLM). The third component comprised a set of *L* random effects, *f*, defined in terms of a set of *L* covariates *z*. Within this framework, a random effect *f*_*l*_ can take a variety of different forms, including unstructured and spatially- and temporally-structured random intercepts and slopes. Together, components one, two, and three can be considered a generalized linear mixed model (GLMM). All latent Gaussian models were fitted and evaluated using the R-INLA package [35] for R statistical computing software [48], a package that facilitates rapid analysis of complex latent Gaussian models on large datasets through use of integrated nested Laplace approximation [47,49].

The 6 models used in this analysis had several characteristics in common. For all 6 models, the dependent variable was relative insecticide use. The independent variables in all 6 models included 7 additive fixed effects, including the (1) proportion of harvested cropland in corn grain and silage, (2) proportion cotton, orchards, and vegetables, (3) proportion soybean and wheat, (4) average farmer net income per hectare, (5) average farm size per county, (6) cumulative annual growing degree days, and (7) proportion of a county in harvested cropland, i.e., landscape simplification, the independent variable of primary interest. Except for the dependent variable, all model variables were centered prior to analysis to aid computation and interpretation of parameter estimates. Default vague prior distributions were used for the intercept, Normal(0, 0), and all fixed effects, Normal(0, 0.001), where the second parameter in each distribution is precision = 1 / variance.

Regarding model specifics, Model 1 was our base model, a simple GLM with an intercept and the 7 continuous fixed effects described above. Model 2 was a GLM that included all terms in Model 1, as well as 2 additional fixed effects: US state, a factor with 47 levels, and year, modeled as a continuous fixed effect. Models 1 and 2 were, essentially, stationary models, where the influences of the 7 common fixed effects were assumed to be consistent over space and time. While Model 1 ignored space and time completely, Model 2 included spatial (state) and temporal (year) fixed effects to allow for additive adjustments to the model intercept, with the possible outcome of reducing residual autocorrelation found in the base model.

Model 3 was a GLM that included all terms in Model 2, as well as three additional interaction terms: state × proportion cropland, year × proportion cropland, and state × year × proportion cropland. The main effects of state and year in Model 3 allowed for additive adjustments to the model intercept. The interactions in Model 3 provided a relatively simple way to explore spatial and temporal heterogeneity in the landscape simplification effect.

Model 4 was a GLMM that included the intercept and all 7 fixed effects of Model 1, an additional continuous fixed effect for year, and 2 random effects to accommodate spatial and temporal structure in the data not accounted for by the fixed effects. With respect to Eq 1, the third term in Model 4 comprised an unstructured random intercept per US county and an unstructured random intercept per year. Default vague prior distributions were used for random intercepts, Log-Gamma(1, 0.00005), where, again, the second parameter is precision.

Model 5 was a GLMM that included the intercept and all 7 fixed effects of Model 1, an additional continuous fixed effect for year, and 3 random effects to accommodate spatial and temporal structure in the data not accounted for by the fixed effects. With respect to Eq 1, the third term in Model 5 comprised an unstructured random intercept per US county, an unstructured random intercept per year, and a spatially (US county) and temporally (year) structured random slope term for proportion cropland, which followed a conditional autoregressive (CAR) specification [50] that evolved across years following a first order autoregressive (AR1) process [51]. In this model and in Model 6, below, we used default prior specifications for both the CAR [Log-Gamma(1, 0.00005)] and AR1 [Log-Gamma(1, 0.00005), rho: Normal(0, 0.15)] components. A GLMM such as Model 5 is often referred to as a spatially varying coefficients (SVC) model [38,39]. Spatially varying coefficients for the landscape simplification effect were calculated per county as the sum of the global fixed effect of proportion cropland and the county random effect of proportion cropland [38].

Model 6, like Model 5, was a GLMM that included the intercept and all 7 fixed effects of Model 1, an additive year fixed affect, and 3 random effects to accommodate spatial and temporal structure in the data. Model 6 was different from Model 5 in that it assumed that the effect of landscape simplification was stationary across space and time. Here, the third term included an unstructured random intercept per US county, an unstructured random intercept per year, and a spatially (US county) and temporally (year) structured random intercept, which followed a CAR specification [50] that evolved across years following an AR1 process [51].

The plausibility of Models 1 through 6 was checked by inspecting histograms of probability integral transform (PIT) values [35]. Coherence between model predictions and observed responses was indicated by a histogram with a shape reasonably close to a uniform distribution [52]. Models were also judged by inspecting maps and empirical variograms of model residuals [53], where residuals were computed as the means from posterior predictive distributions minus observed values [54] and pairwise distances were estimated from the geographic coordinates of county centroids.

The overall performances of Models 1 through 6 were compared using the Deviance Information Criterion (DIC) [55], a generalization of Akaike’s Information Criterion (AIC) [56]. Using this metric, the model that best balances short-term prediction and parsimony, the DIC-best model, is the one with the lowest DIC value. The ΔDIC value for a given model is the difference in DIC units between the best model and that of a given model. Models with ΔDIC < 5 are generally considered competitors with the best model. Models with 5 < ΔDIC < 10 are considered poor competitors with the best model. Models with ΔDIC > 10 can be ruled out as alternatives to the best model [54]. Inference about model parameters was based on posterior marginal distributions from the best or set-of-best models. Every detail of model specification, estimation, and evaluation can be found in the computing code that is provided, along with the full dataset, in S1 Code and Data.

## Results

**Fig 1:**
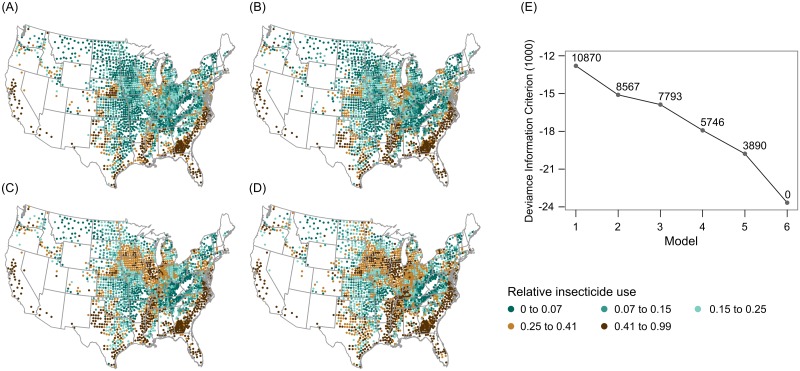
Observed relative insecticide use across the conterminous US during 1997, 2002, 2007, and 2012 (A-D, respectively), as well as DIC and ΔDIC values (E) for the six predictive models evaluated in this study. Relative insecticide use (A-D) is the proportion of harvested cropland in a county treated with insecticide. DIC values on the y-axis (E) have been divided by 1000. Untransformed ΔDIC values are shown as point labels (E).

From 1997 through 2012, the proportion of harvested cropland treated with insecticide, or relative insecticide use, averaged 0.249 across the US, although county values ranged from 0 to nearly 1 (Fig 1A–1D). Crop composition variables—proportions of harvested cropland in (1) corn grain and silage, (2) cotton, orchards, and vegetables, and (3) soybean and wheat—also ranged widely, from 0 to nearly 1 (S1–S3 Figs). Mean annual net income of farmers averaged $US 610 per harvested hectare, but ranged from losses of $US -2,323 to gains of $US 16,923 per harvested hectare (S4 Fig). The amount of harvested cropland per farm operator averaged 1.196 km^2^, though it ranged from 0.053 km^2^ to 13.825 km^2^ across counties in this study (S5 Fig). Accumulated annual growing degree days ranged from 536 in the northcentral US to 6,051 in the Southeast (S6 Fig). Finally, the proportion of a county in harvested cropland averaged 0.287, with some counties having relatively little while, for others, it was the dominant land cover type (S7 Fig).

The set of 6 models considered in this analysis had several characteristics in common (Tables 1 and 2). For example, the effect of the proportion of cropland in corn on relative insecticide use was fairly consistent across the 6 models, with posterior mean slopes ranging from 0.396 to 0.471. The proportion of cropland in orchards, vegetables, and cotton also had a fairly consistent effect across models, with mean slopes ranging from 0.605 to 0.754. The effect of the proportion of cropland in soybean and wheat was consistently low across the models, with slopes ranging from 0.022 to 0.053. The effect of mean farm size was consistently positive across the 6 models, with mean slope values ranging from 0.012 to 0.031. The effect of annual growing degree days was also consistently positive across the models, with mean slopes ranging from 0.030 to 0.093. Other fixed effects varied across the 6 models. For example, the effect of farmer net income was null in some models and positive in others. And mean slopes for the proportion of a county in cropland ranged widely, from -0.119 to positive 0.304, making inference about the strength and direction of the association between landscape simplification and insecticide use highly dependent upon model choice.

**Table 1:** Select Parameter Estimates from Three of Six Models of Relative Insecticide Use across the Conterminous US from 1997 through 2012.

	Model 1	Model 2	Model 3
**Posterior mean estimates (2.5, 97.5 percentiles) of fixed effects**			
Model intercept	0.249 (0.247, 0.252)	0.271 (0.252, 0.290)	0.331 (0.279, 0.384)
Proportion cropland in corn grain and silage	0.471 (0.452, 0.490)	0.463 (0.441, 0.486)	0.450 (0.427, 0.473)
Proportion in orchards, vegetables, cotton	0.754 (0.735, 0.772)	0.668 (0.648, 0.688)	0.646 (0.625, 0.667)
Proportion in soybean and wheat	0.053 (0.039, 0.067)	0.047 (0.033, 0.062)	0.048 (0.033, 0.063)
Annual net income per harvested hectare	0.009 (0.006, 0.011)	-0.001 (-0.003, 0.001)	0.000 (-0.002, 0.002)
Square kilometers per farm operator	0.031 (0.028, 0.034)	0.026 (0.023, 0.029)	0.022 (0.019, 0.025)
Annual accumulated growing degree days	0.079 (0.076, 0.082)	0.075 (0.067, 0.083)	0.072 (0.065, 0.080)
Proportion of county in harvested cropland	-0.119 (-0.137, -0.101)	-0.002 (-0.021, 0.016)	0.304 (0.046, 0.562)
Year	.	0.004 (0.003, 0.004)	0.004 (0.003, 0.004)
State	.	*	*
Year, state, and proportion harvested interactions	.	.	*
**Posterior mean standard deviations (2.5, 97.5 percentiles) associated with random effects**			
Model error	0.122 (0.120, 0.124)	0.108 (0.106, 0.109)	0.103 (0.101, 0.104)
Random intercept, county (exchangeable)	.	.	.
Random intercept, year (exchangeable)	.	.	.
Random intercept, county (CAR) within year (AR1)	.	.	.
Random slope, cropland, county within year	.	.	.
**Posterior mean estimates (2.5, 97.5 percentiles) for rho**			
Rho, random intercept AR1	.	.	.
Rho, random slope AR1	.	.	.
**Model selection scores**			
Deviance Information Criterion (DIC)	-12,798	-15,102	-15,875
ΔDIC	10,870	8,567	7,793

Note: Periods indicate the absence of a variable in a model, and asterisks indicate too many coefficients to list.

**Table 2:** Select Parameter Estimates from Three of Six Models of Relative Insecticide Use across the Conterminous US from 1997 through 2012.

	Model 4	Model 5	Model 6
**Posterior mean estimates (2.5, 97.5 percentiles) of fixed effects**			
Model intercept	0.249 (0.239, 0.260)	0.258 (0.247, 0.268)	0.265 (0.255, 0.275)
Proportion cropland in corn grain and silage	0.454 (0.428, 0.480)	0.396 (0.368, 0.423)	0.398 (0.365, 0.430)
Proportion in orchards, vegetables, cotton	0.689 (0.662, 0.716)	0.641 (0.613, 0.670)	0.605 (0.575, 0.635)
Proportion in soybean and wheat	0.022 (0.003, 0.042)	0.023 (0.003, 0.044)	0.041 (0.019, 0.063)
Annual net income per harvested hectare	0.011 (0.008, 0.014)	0.011 (0.008, 0.014)	0.004 (0.001, 0.007)
Square kilometers per farm operator	0.030 (0.026, 0.033)	0.023 (0.019, 0.027)	0.012 (0.007, 0.016)
Annual accumulated growing degree days	0.077 (0.072, 0.082)	0.093 (0.087, 0.100)	0.030 (0.013, 0.046)
Proportion of county in harvested cropland	-0.103 (-0.129, -0.078)	-0.049 (-0.083, -0.016)	0.050 (0.022, 0.078)
Year	0.004 (0.002, 0.005)	0.002 (0.000, 0.004)	0.005 (0.003, 0.007)
State	.	.	.
Year, state, and proportion harvested interactions	.	.	.
**Posterior mean estimates (2.5, 97.5 percentiles) of fixed effects**			
Model error	0.084 (0.082, 0.085)	0.073 (0.071, 0.074)	0.054 (0.052, 0.056)
Random intercept, county (exchangeable)	0.087 (0.084, 0.090)	0.069 (0.066, 0.073)	0.005 (0.003, 0.010)
Random intercept, year (exchangeable)	0.008 (0.005, 0.020)	0.007 (0.004, 0.018)	0.008 (0.005, 0.019)
Random intercept, county (CAR) within year (AR1)	.	.	0.150 (0.146, 0.155)
Random slope, cropland, county within year	.	0.443 (0.407, 0.478)	.
**Posterior mean estimates (2.5, 97.5 percentiles) for rho**			
Rho, random intercept AR1	.	.	0.708 (0.676, 0.739)
Rho, random slope AR1	.	0.787 (0.724, 0.832)	.
**Model selection scores**			
Deviance Information Criterion (DIC)	-17,922	-19,779	-23,668
ΔDIC	5,746	3,890	0

Note: Periods indicate the absence of a variable in a model, and asterisks indicate too many coefficients to list.

**Fig 2:**
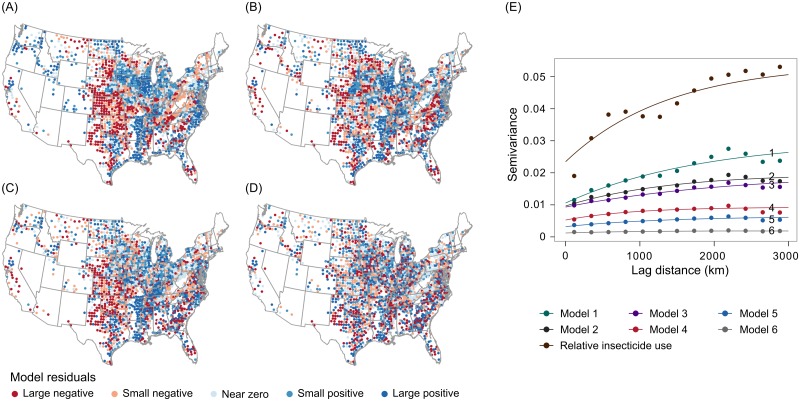
Maps of spatial structure in 2012 residuals from Model 1, Model 3, Model 5, and Model 6 (A-D, respectively), and empirical variograms with Matérn fits (E) for residuals of those models, as well as from Model 2 and Model 4, and the raw observed values of relative insecticide use. Only 2012 data are shown, but very similar patterns occurred across all years.

Choosing a best model for inference is not advised when competing models provide similar fits to the data but support different conclusions [57]. In this study, however, there were substantial differences in model fit across the 6 models. DIC values decreased progressively from Model 1 (-12,798) through Model 6 (-23,668), with Model 6 having the lowest DIC value (Tables 1 and 2, Fig 1E). ΔDIC values for Models 1 through 5 (3,890 ≤ ΔDIC ≤ 10,870) were much larger than the 10 units that would indicate multiple competing models (Tables 1 and 2). Similar patterns were evident when looking at other characteristics of model fit. For example, the standard deviations of model residuals decreased considerably from Model 1 through Model 6 (Tables 1 and 2, Model error). We also noted clear spatial patterning in the residuals of Models 1 through 5. This was obvious both in maps (Fig 2A–2D) and empirical variograms (Fig 2E) of model residuals. We expected to see spatial structure in the residuals of Model 1 (Fig 2A and 2E), based on results from previous studies [24]. It was surprising, however, that autocorrelation was also strong in the residuals of Model 2, which had a spatial fixed effect; Model 3 (Fig 2B and 2E), which had a spatial fixed effect and allowed for spatial variation in the proportion cropland effect through interaction terms; Model 4; and Model 5 (Fig 2C and 2E), the SVC model with a spatially-structured random slope for the proportion cropland effect. The only model that did not have pronounced spatial structure in residuals was Model 6 (Fig 2D and 2E), with a spatially-structured random intercept. Rho estimates from Model 5 (0.787) and Model 6 (0.708) indicated that relative insecticide use also exhibited temporal structure not explained by the fixed effects (Table 2).

**Fig 3:**
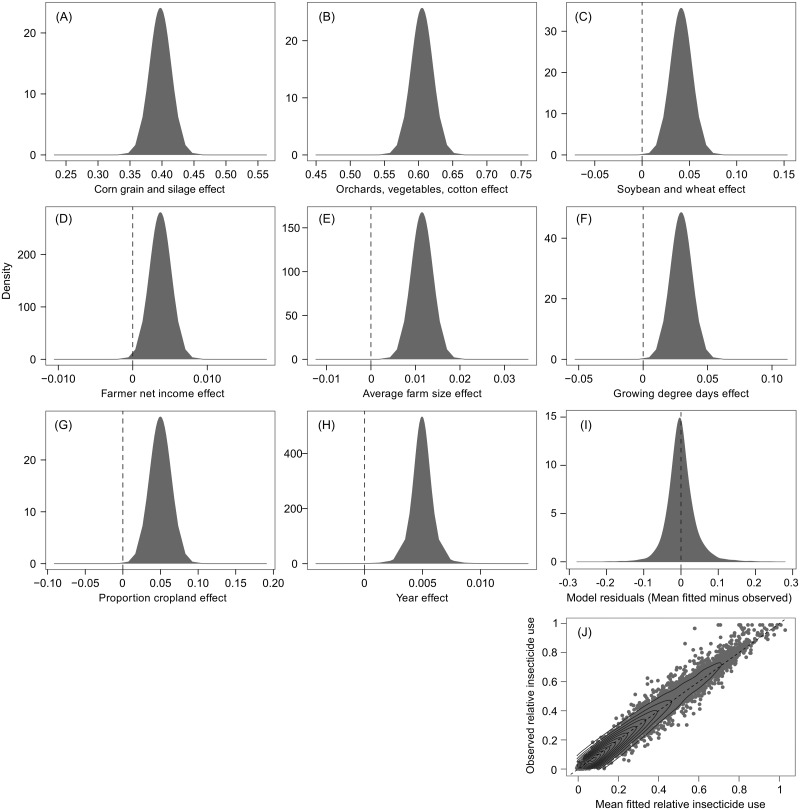
Posterior marginal distributions for fixed effects from Model 6, along with a histogram of model residuals and a plot of predicted versus observed relative insecticide use. Vertical dashed lines (A-I) highlight parameter values of 0. The diagonal dashed line (J) indicates unity. Contour lines (J) illustrate data density.

Given markedly better performance, we used posterior marginal distributions for parameter estimates from Model 6 to make inferences about variables associated with insecticide use. For example, Model 6 included a significant intermediate effect of corn in agricultural landscapes (Table 2, Fig 3A). The mean parameter estimate for the corn effect (0.398) suggested that, in an average landscape, all else being equal, an increase from 40% to 80% in the proportion harvested cropland in corn was associated with an increase in relative insecticide use, which ranges from 0 to 1.00, of 0.16. There was a strong significant effect of cotton, orchards, and vegetables (Table 2, Fig 3B). The mean effect (0.605) suggested that an increase from 10% to 30% in the proportion harvested cropland in cotton, orchards, and vegetable production was associated with an increase in relative insecticide use of 0.12. Model 6 had a small but statistically significant effect of soybean and wheat (Table 2, Fig 3C). This effect (0.041) suggested that an increase in the proportion soybean and wheat from 40% to 80% was associated with an increase in relative insecticide use of 0.02.

Model 6 also included statistically significant associations between relative insecticide use and farmer net income and average farm size (Table 2, Fig 3D and 3E). The posterior mean effect of farmer net income was 0.004, indicating that each increase of $US 1,000 per hectare was associated with an increase in relative insecticide use of 0.004. The posterior mean effect of average farm size was 0.012, indicating that each increase in average farm size of 100 ha was associated with an increase in relative insecticide use of 0.01. Model 6 also included a significant association between annual growing degree days and relative insecticide use (Table 2, Fig 3F). The posterior mean effect, 0.030, indicated that each increase of 1,000 GDD was associated with an increase in relative insecticide use of 0.03. Model 6 suggested that relative insecticide use in the US has been increasing since 1997 (Table 2, Fig 3H). The mean of the posterior distribution for the year effect was 0.005, indicating that, after accounting for temporal changes in other model variables, the proportion of cropland receiving insecticide increased by roughly 0.08 over the 15 years of the study.

Finally, after accounting for all other covariates, Model 6 had a significant positive association between landscape simplification and relative insecticide use (Table 2, Fig 3G). The marginal posterior distribution for the proportion cropland effect gave an 80% credible interval of 0.032–0.068 and a 95% credible interval of 0.022–0.077, and indicated that a slope ≤ 0 had a probability of 0.0002 (Fig 3G). Histograms of PIT values and model residuals (Fig 3I) and plots of mean predicted versus observed values (Fig 3J) indicated that Model 6 provided a sufficient fit to the data. Multicollinearity was not a problem, as pairwise correlations between fixed effects ranged from -0.32 to 0.65. The posterior mean effect of proportion cropland in Model 6 was 0.050, indicating that, in an average agricultural county, all else being equal, an increase in proportion cropland from 20% to 80% was associated with an increase in relative insecticide use of 0.03.

## Discussion

Results from this analysis suggest that, after accounting for several other important variables, as well as unidentified spatial and temporal structure in the data, there was an overall positive relationship between landscape simplification and insecticide use across the conterminous US between 1997 and 2012. The overall positive relationship between landscape simplification and insecticide use supports predictions from the chain of hypotheses outlined in the introduction [1–4]. The estimated effect (0.050) is consistent with results from Meehan et al. [5] and Meehan and Gratton [24], who used similar spatial modeling approaches and reported very similar landscape simplification coefficients (between 0.04 and 0.10) for the Midwestern US. The association contrasts with those from other studies in the Midwestern US and across the United States that used different modeling techniques [23,27] and concluded that the association was too variable in space and time to allow generalization.

A general positive association between landscape simplification and insecticide use was inferred from parameter estimates from a stationary spatiotemporal statistical model (Model 6), which fit the data better than a stationary alternative with spatial and temporal fixed effects (Model 2), and nonstationary alternatives that included a model with interactions between spatial and temporal fixed effects and proportion cropland (Model 3) and an SVC model (Model 5). The superior fit of Model 6 was indicated by a model DIC value that was 3,890 DIC units lower than the next best model. Relative fit was also judged by the degree to which models yielded residuals without considerable spatial and temporal autocorrelation. Spatial patterns in model residuals, in particular, can indicate that important patterns and processes are not sufficiently represented by models [58–60]. Numerous simulation studies have shown that the consequences of this form of model misspecification include biased parameter estimates and unreliable hypothesis tests [41,43–45,61]. Indeed, strong spatial autocorrelation in this study (Fig 2A–2C and 2E) was associated with considerably different parameter estimates for some variables. For example, estimates for the proportion cropland effect ranged from -0.119 to 0.304 across Models 1 through 5 (Tables 1 and 2).

Regarding other covariates in this analysis, the intermediate corn effect (0.398) was similar to those reported in other studies, which generally ranged from 0.23 to 0.46 [5,23–25,27]. The large effect of cotton, fruit, and vegetables (0.605) was among those reported in other studies (0.48–0.93) and reflects the historical high rates of insecticide use in those crops [5,23,24,27]. The positive relationships between insecticide use, farmer income, and average farm size were similar to those reported in recent studies [5,23–25,27], and could have arisen for several interrelated reasons. For example, higher-earning producers could have more to lose from pest-related reductions in crop productivity or value, and could have increased capacity to purchase insecticides and hire applicators [5]. Larsen et al. [27] described possible economic and ecological mechanisms behind the association between insecticide use and farm size. For example, larger farms may have a greater capacity to invest in insecticide and application equipment, and may coordinate pest control activities over larger areas [27]. Alternatively, larger farm operations might work larger fields, with ecological implications related to increased pest colonization and decreased natural enemy activity [27].

Model 6 suggested a substantial increase in relative insecticide use over the 15 years of the study. This increase, also reported elsewhere [24,25,62], occurred despite widespread adoption of transgenic corn in the US, which is generally expected to reduce insecticide use [63]. Future research could explore whether a recent increase reflects a response by farmers [62] to evolved resistance by insect pests to *Bacillus thuringiensis* toxins in corn [64], or to increased populations of secondary non-target pests [65]. As reported by Yang et al. [25], the proportion of harvested cropland receiving insecticide treatment was positively associated with cumulative annual growing degree days in Model 6. Yang et al. [25] suggested that a positive relationship might be expected based on the positive effect of temperature on pest growth rates [66]. However, the same logic applies to the mutualists and natural enemies of pests [67], making straightforward predictions difficult [68]. It is tempting to extend the relationship between insecticide use and GDD to say something about future insecticide use on a warming planet. The naïve prediction is that increased global temperatures will bring increased insecticide use. However, given the complexity of climate-crop-pest-natural enemy systems [69], and the uncertainty in predictions about future temperature, precipitation, nutrient pollution, and atmospheric gas concentrations [70], this simple prediction could easily be incorrect, or even correct for the wrong reasons.

To determine the relative strength of the different model covariates, standardized coefficients were computed for all fixed effects in Model 6. This exercise indicated that the fixed effects fell into three general groups. The group with the largest effects on insecticide use included the crop composition variables, specifically the proportion of harvested cropland in corn (standardized coefficient = 0.076) and the proportion in cotton, orchards, and vegetables (0.098). The group with intermediate effects on insecticide use included the proportion of harvested cropland in soybean (0.010), average farm size (0.014), annual growing degree days (0.027), and the proportion of a county in harvested cropland (0.012), or landscape simplification. The group with the smallest effect on insecticide use included farmer net income (0.004). As discussed in Meehan et al. [5] and Meehan and Gratton [24], it is not possible to know from these data if the modest association between landscape simplification and insecticide use was due to modest effects of landscape simplification on pest pressure, or to low sensitivity of insecticide application to pest pressure. Indeed, it is common for growers to base insecticide application decisions on calendar day, with little information on pest abundance [20].

In conclusion, our analysis suggested an overall positive relationship between landscape simplification and insecticide use across the conterminous US from 1997 through 2012. The overall positive trend is consistent with the hypothesis that landscape simplification commonly affects the ecology of crop pests or their natural enemies in ways that lead to pest pressure with meaningful agronomic consequences. While the trend is consistent with predictions, it does not confirm mechanisms. Opportunities to study mechanisms at large spatial and temporal scales have been limited. Ideally, additional information on pests, natural enemies, and crop damage would be available to simultaneously evaluate multiple aspects of this causal model. Meehan et al. [5] were able to link landscape characteristics to pest abundance, and then to insecticide use, but only for the Midwestern US during a single year. Exploring links between landscape characteristics, pest movement, natural enemy activity, and insecticide use at larger spatial and temporal scales will require considerable creativity and resources.

## Supporting Information

S1 Fig Maps depicting the proportion of harvested cropland in corn for grain and silage. (TIF)

S2 Fig Maps depicting the proportion of harvested cropland in orchards, vegetable, and cotton production. (TIF)

S3 Fig Maps depicting the proportion of harvested cropland in soybean and wheat production. (TIF)

S4 Fig Maps depicting the spatial distribution of farmer annual net income. (TIF)

S5 Fig Maps depicting the spatial distribution of average farm size. (TIF)

S6 Fig Maps depicting the spatial distribution of annual growing degree days. (TIF)

S7 Fig Maps depicting the spatial distribution of proportion of county in harvested cropland, i.e., landscape simplification. (TIF)

S8 Fig Maps depicting the spatial distribution of residuals from Model 6. (TIF)

S1 Code and Data R code and data necessary to reproduce the full analysis. (ZIP)
