# Supplementary figures and images for: A Landscape View of Agricultural Insecticide Use across the Conterminous US from 1997 through 2012
Source: PLoS One. 2016 Nov 30;11(11):e0166724. doi: 10.1371/journal.pone.0166724 (PMC5130224; doi:10.1371/journal.pone.0166724)

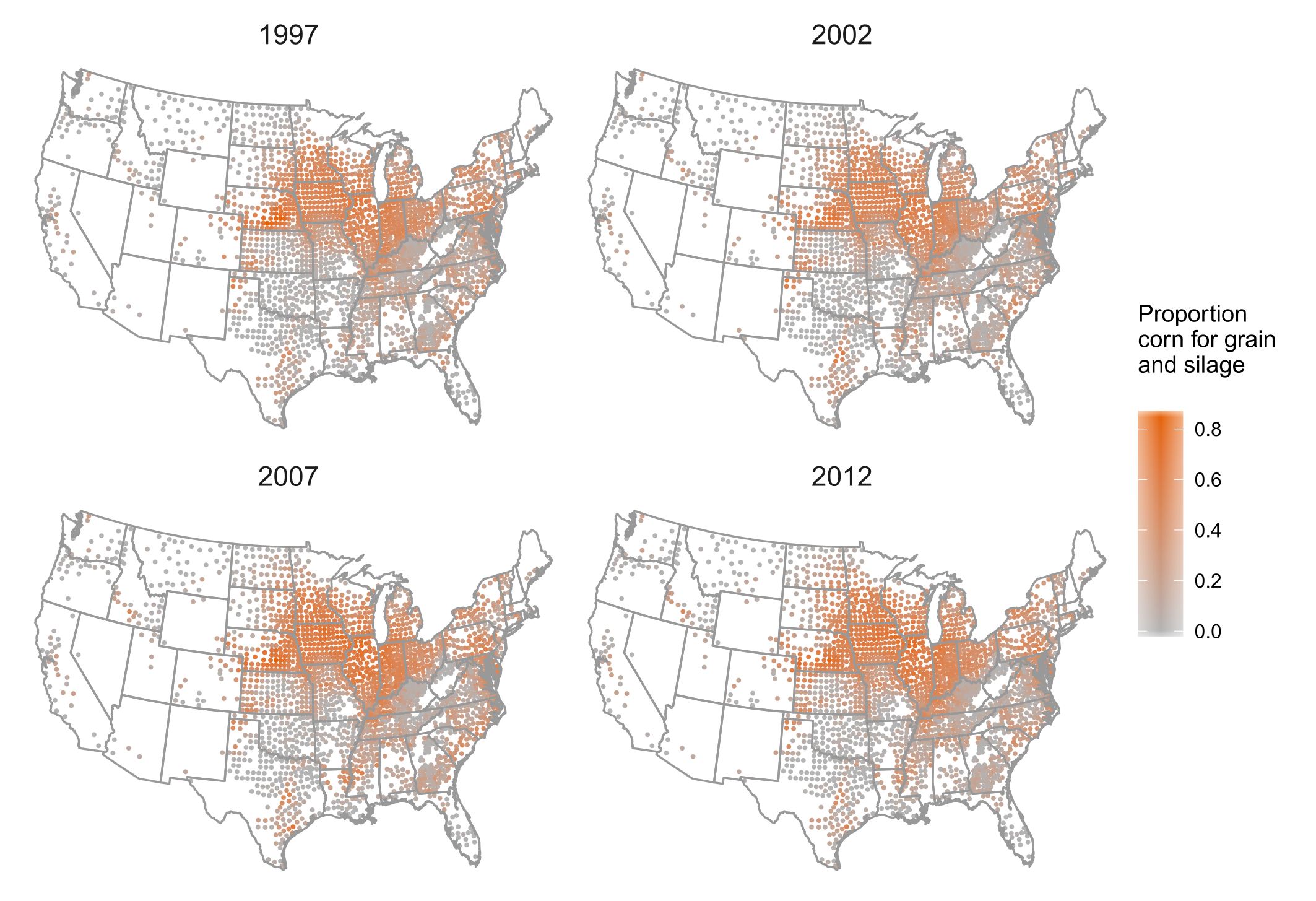

Supplement: S1 Fig — (TIF) [file pone.0166724.s001.tif]

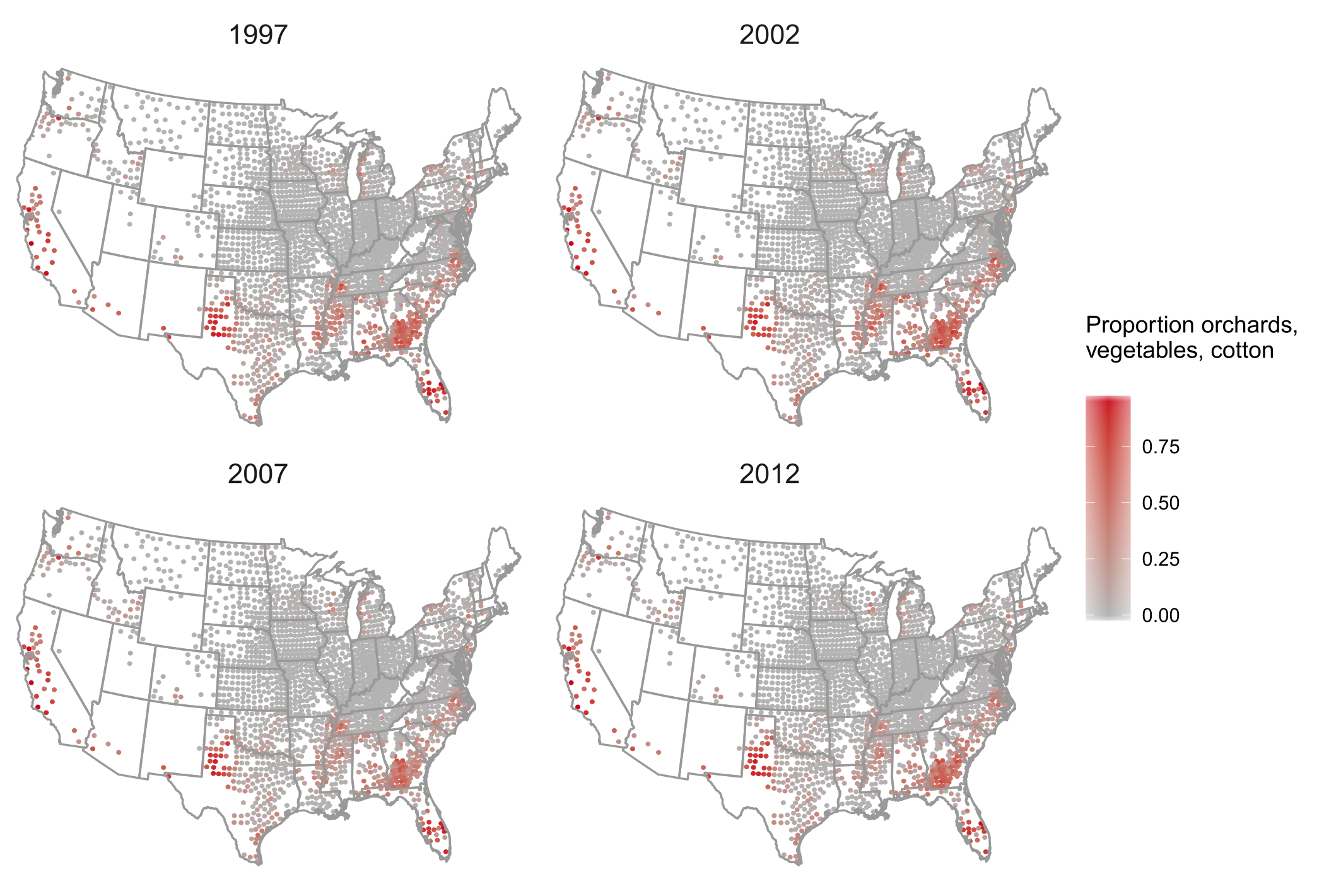

Supplement: S2 Fig — (TIF) [file pone.0166724.s002.tif]

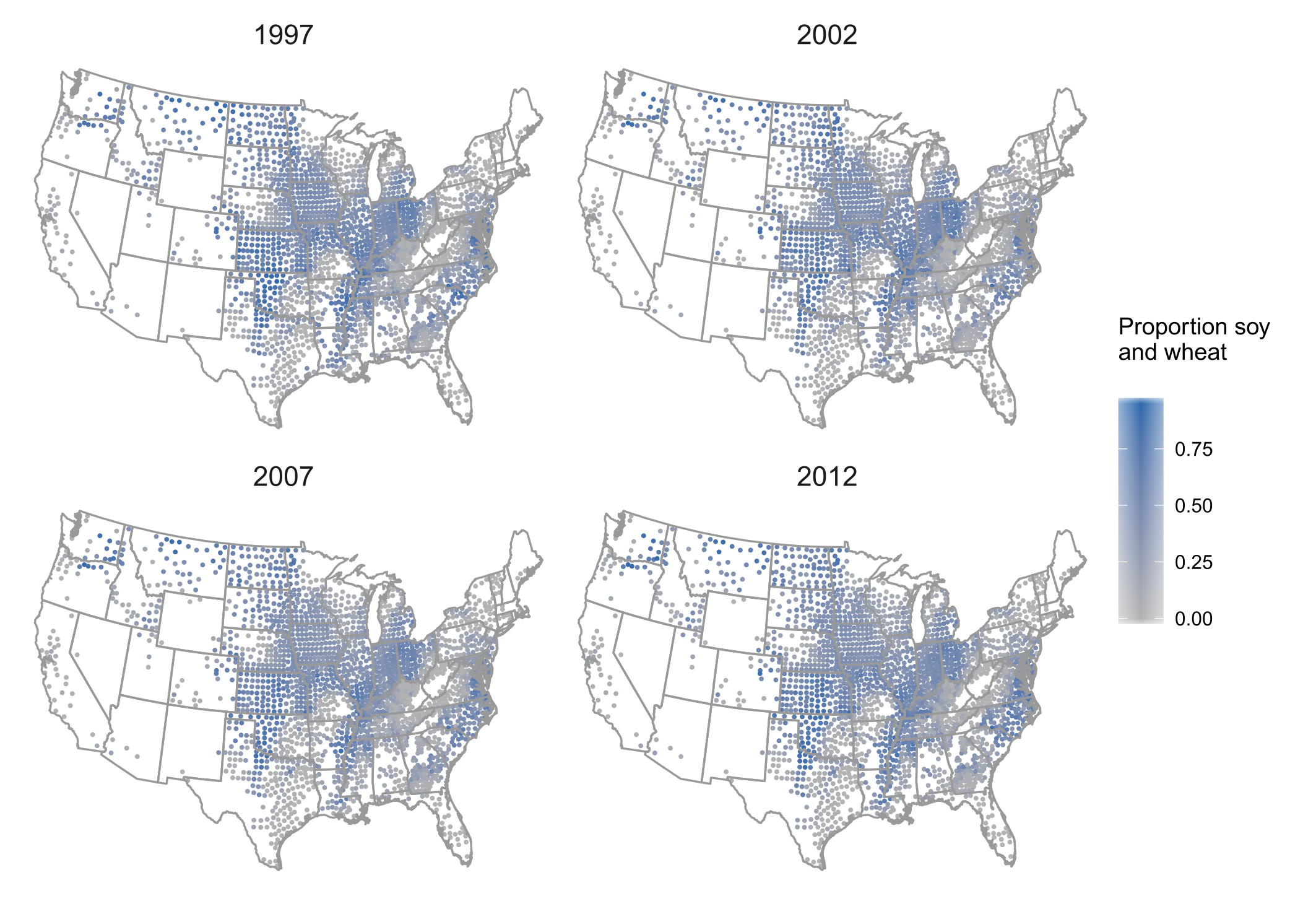

Supplement: S3 Fig — (TIF) [file pone.0166724.s003.tif]

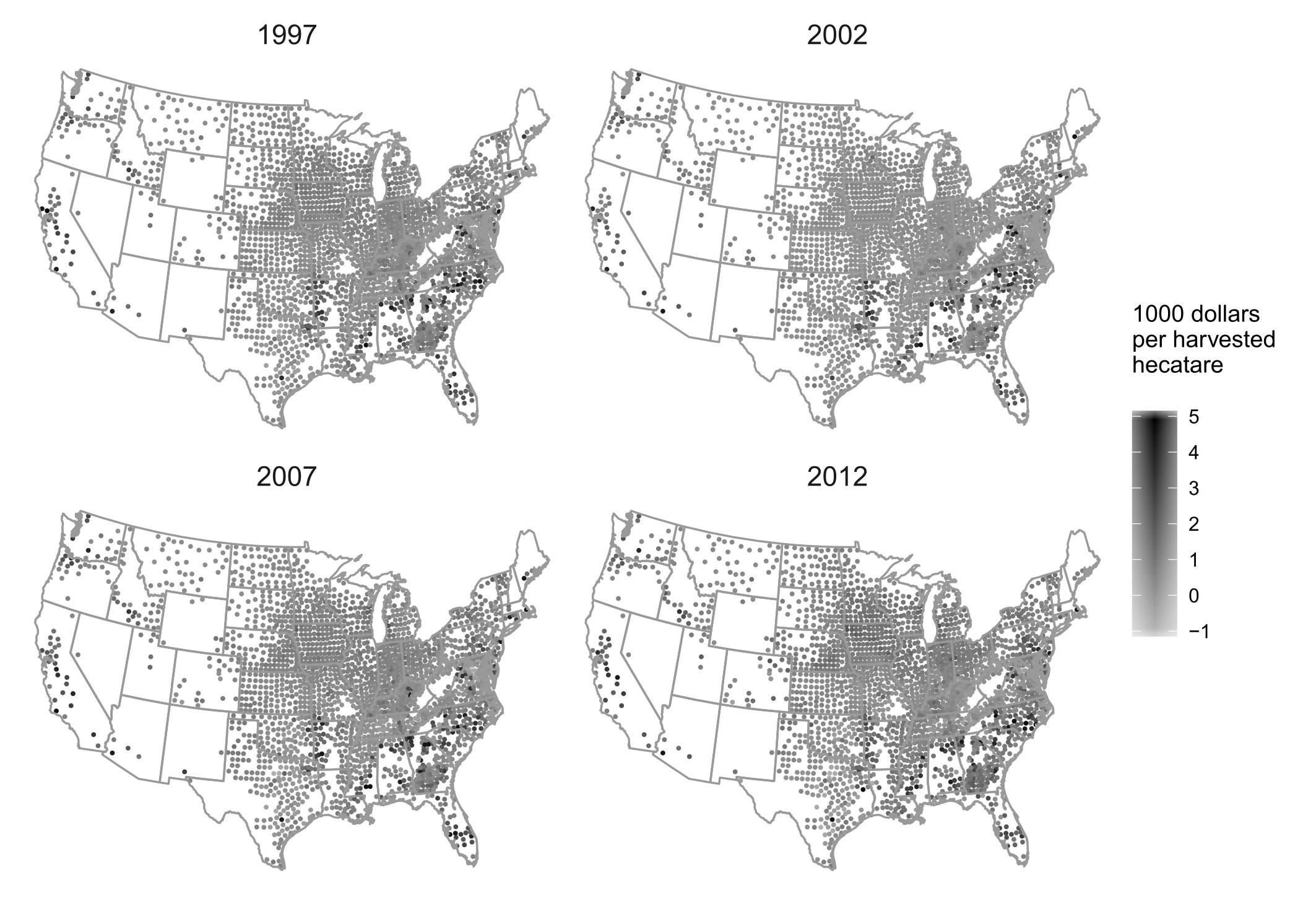

Supplement: S4 Fig — (TIF) [file pone.0166724.s004.tif]

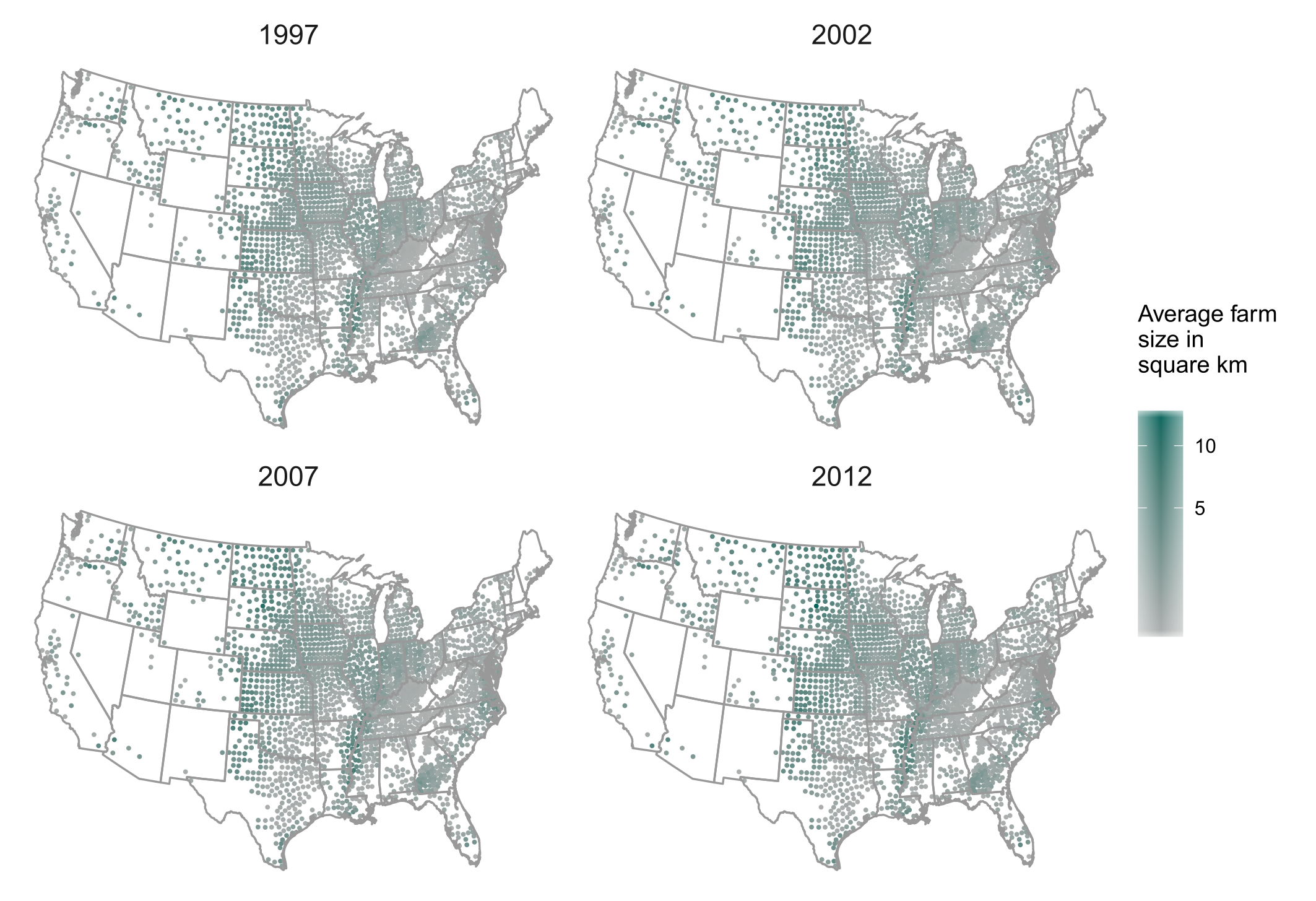

Supplement: S5 Fig — (TIF) [file pone.0166724.s005.tif]

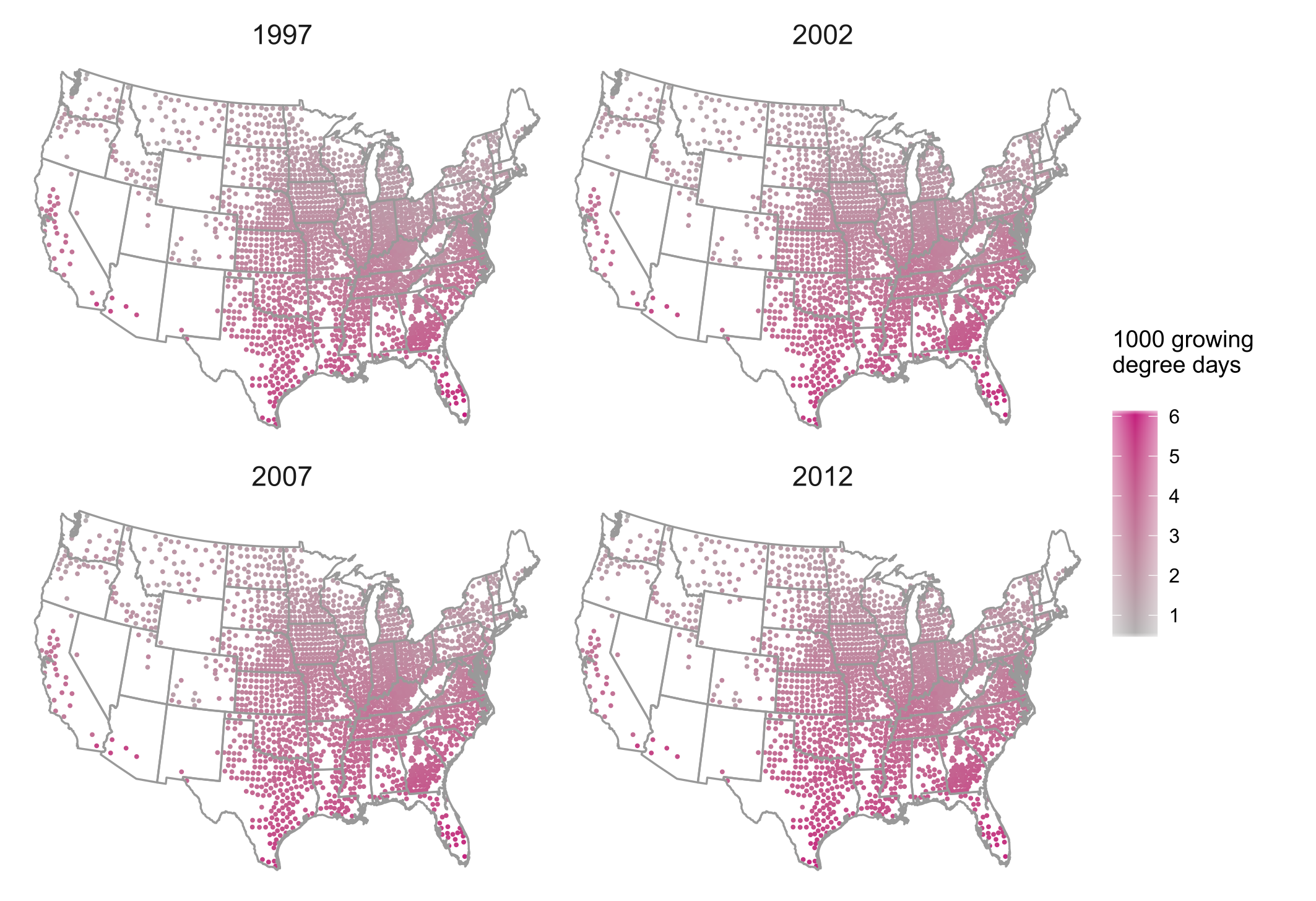

Supplement: S6 Fig — (TIF) [file pone.0166724.s006.tif]

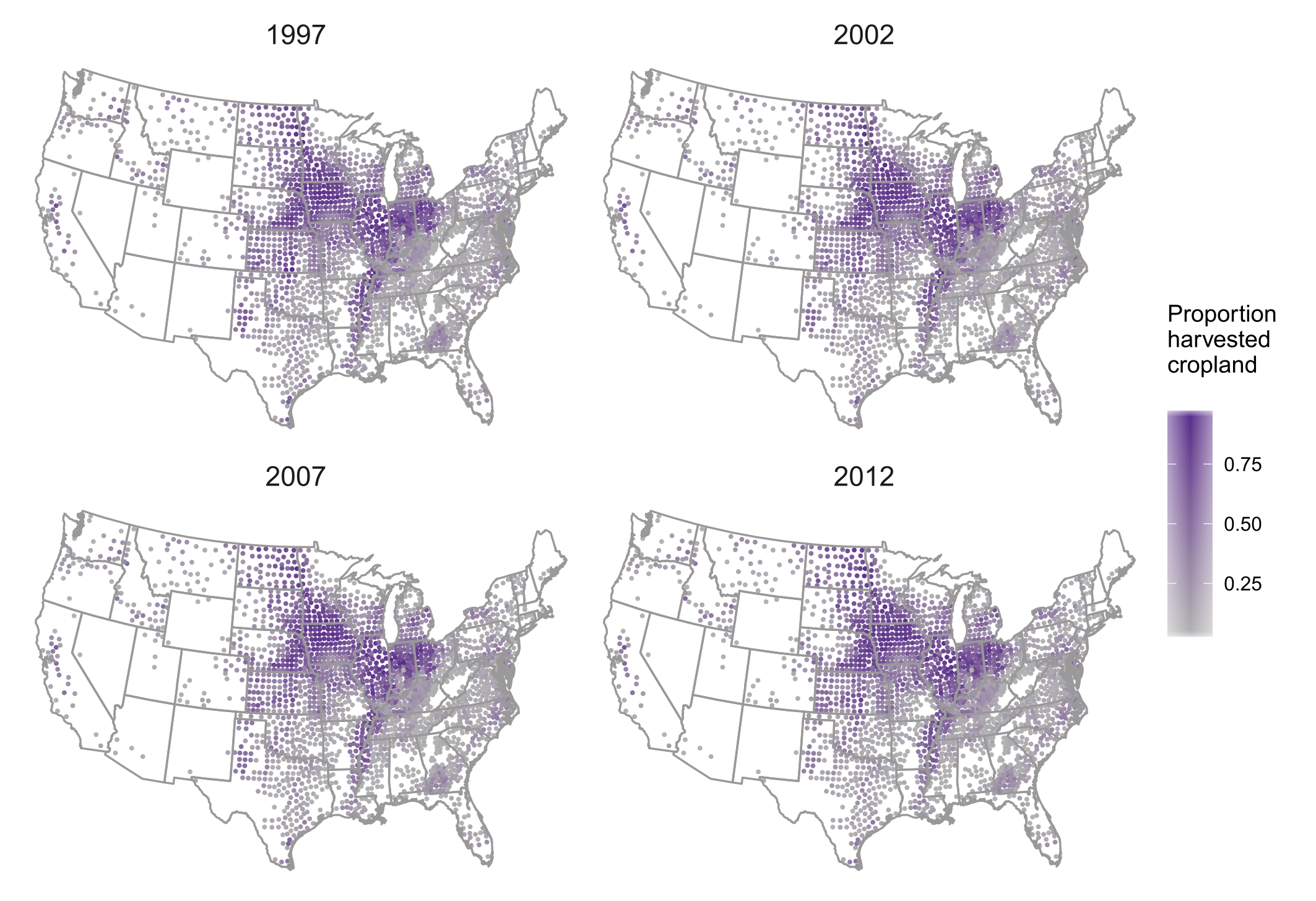

Supplement: S7 Fig — (TIF) [file pone.0166724.s007.tif]

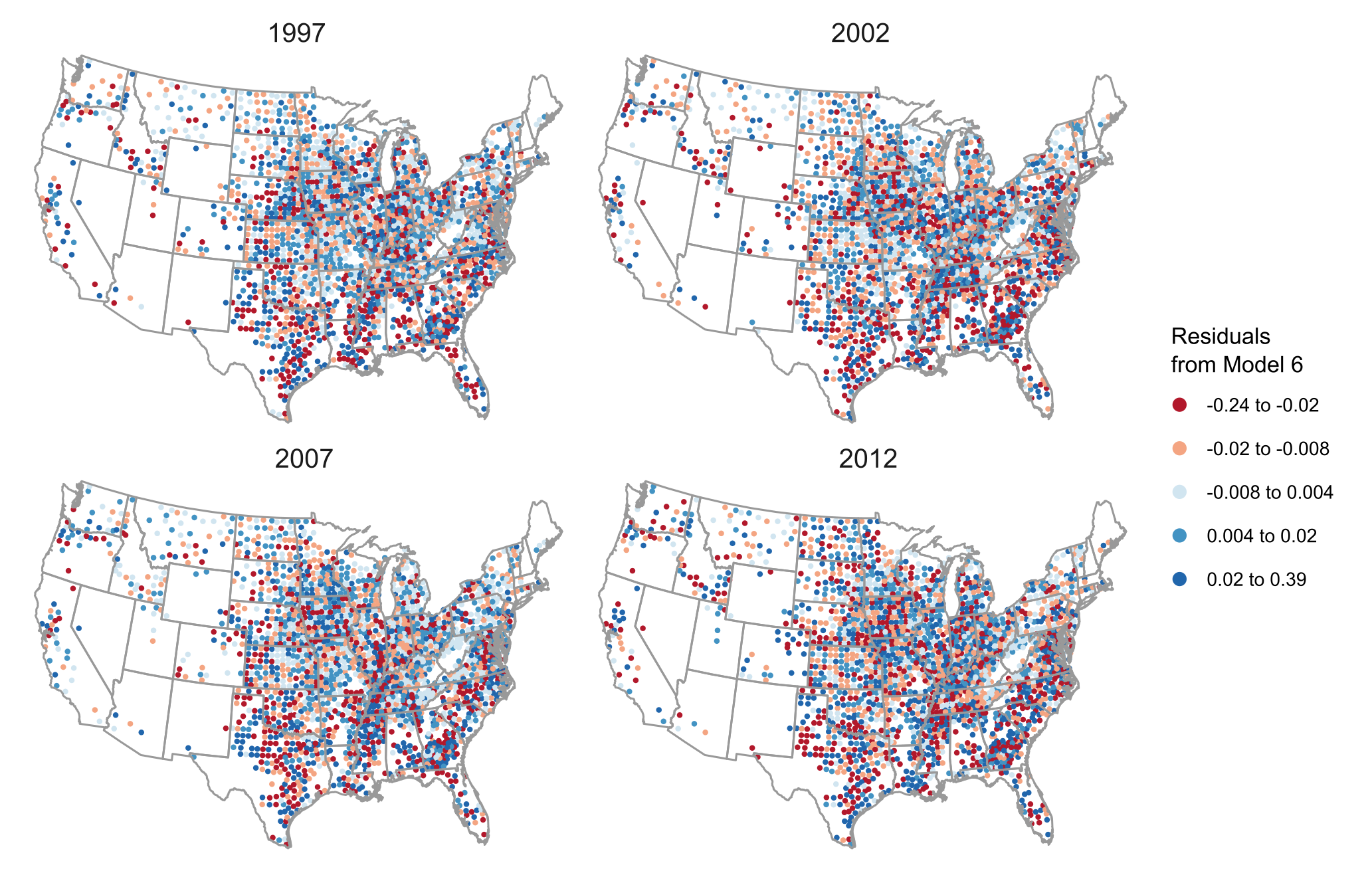

Supplement: S8 Fig — (TIF) [file pone.0166724.s008.tif]
